# Heatstroke knowledge and predictors among Hajj health volunteers in Saudi Arabia: a cross-sectional study

**DOI:** 10.7717/peerj.20816

**Published:** 2026-02-19

**Authors:** Fatmah Alamoudi, Halah Almulla, Magda Yousif, Nouf Alnaimi, Adil Abdalla, Mahmoud Abdel Hameed Shahin, Faizan Kashoo

**Affiliations:** 1 Nursing Department, Prince Sultan Military College of Health Sciences, Dhahran, Saudi Arabia; 2 Cardiac Department, King Fahad Military Medical Complex (KFMMC), Dhahran, Eastern Province, Saudi Arabia; 3 Department of Physical Therapy and Health Rehabilitation, College of Applied Medical Sciences, Majmaah University, Majmaah, Riyadh, Saudi Arabia

**Keywords:** Heat stroke, Hajj health volunteers, Knowledge assessment, Predictors of awareness, Saudi Arabia

## Abstract

**Background:**

Heatstroke is a life-threatening condition resulting from prolonged exposure to high temperatures or intense physical activity, especially during the summer. Pilgrims performing Hajj are particularly vulnerable because of factors such as advanced age, chronic health conditions, and failure to follow safety guidelines. Health volunteers play a key role in prevention and response.

**Aim:**

This study aimed to assess health volunteers’ knowledge and identify predictors of such knowledge.

**Methods:**

A cross-sectional study was conducted among 772 health volunteers during Hajj 2024 in Saudi Arabia. A self-designed questionnaire was developed and utilized to assess participants’ knowledge of heatstroke and to identify predictors of such knowledge. Data were collected electronically and analyzed *via* Jeffrey’s Amazing Statistics Program (JASP).

**Results:**

Analysis revealed that (*n* = 576) 74.6% of health volunteers had satisfactory heatstroke knowledge, though critical gaps persisted. While recognition of specific symptoms (*e.g.*, lethal temperatures >39.5 °C; 90.8%) and preventive measures was strong, pathophysiological understanding was poor, with only 5.2% correctly interpreting body temperature thresholds. Logistic regression identified significant predictors: a medical educational background (odds ratio (OR) = 4.51, *p* < .001), employment (OR = 2.45, *p* < .001), and previous first aid training (OR = 2.51, *p* = .003) increased odds of satisfactory knowledge. Significant regional disparities existed, with the Middle Region associated with higher odds (OR = 2.14, *p* = .019) and the Southwest Region with lower odds (OR = 0.31, *p* = .004).

**Conclusion:**

In conclusion, this study found that while a majority of Hajj health volunteers possess satisfactory heatstroke knowledge, significant and systematic disparities exist. The findings robustly demonstrate that knowledge is not uniformly distributed but is significantly predicted by educational background, occupational status, and geographic region. To ensure a uniformly high standard of care, preparedness initiatives must be strategically refined. Resources should be deliberately channeled into enhancing the training of non-medical volunteers and addressing the pronounced knowledge gaps in underperforming regions, thereby fortifying the overall resilience of the health volunteer system against the formidable threat of heatstroke.

## Introduction

Population exposure to heat is increasing due to climate change (Klein & Anderegg, 2021). The number of people exposed to extreme heat is increasing worldwide due to global warming. Since 1850, the last three decades have been measured as warmer than any other decade. Increased intensity, duration, and frequency of extreme heat events resulted in increased incidence rates of heat-related illnesses (Faurie et al., 2022). Heatstroke is a serious medical emergency with a high mortality rate (Adnan Bukhari, 2023). Heatstroke occurs when the body’s temperature regulation system fails because of excessive environmental heat alone or combined with extreme physical exertion (Wang et al., 2024). This results in a dangerous elevation in core body temperature that exceeds 40 °C, leading to central nervous system dysfunction manifested by delirium, confusion, and coma (Bouchama et al., 2022). Heatstroke can be classified as classic or exertional. Any exposure to extreme heat and environments with high temperatures can increase the risk of heatstroke. However, some people in the community are at greater risk of experiencing adverse outcomes. Heat can have profound effects on senior adults, children, pregnant women, and people with chronic health conditions such as cardiovascular disease and respiratory, renal, or psychiatric disease (Ndlovu & Chungag, 2024). While predictable, the adverse effects of heatstroke are mainly preventable with public health awareness and multisectoral policies and interventions (Sorensen & Hess, 2022). The World Health Organization and World Meteorological Organization jointly released their first comprehensive workplace heat stress report in August 2025, documenting that worker productivity decreases by 2–3% for each degree above 20 °C and emphasizing risks including heatstroke, kidney dysfunction (Kravets et al., 2023), and neurological disorders (Yoneda et al., 2024). The International Labour Organization reported in July 2024 that excessive heat affects over 2.4 billion workers globally, causing 22.85 million occupational injuries annually and 4,200 deaths in 2020 (Parsons et al., 2022). While laborers often develop robust heat-acclimatization and tolerate thermal stress effectively, the predominantly elderly and medically vulnerable cohort of Hajj pilgrims exhibits comparatively lower tolerance to heat exposure during the pilgrimage.

Hajj is a religious mass gathering annually in the hot, arid climate of Makkah in the Kingdom of Saudi Arabia. Over two million Muslim pilgrims from different ethnic groups from all over the world gather every year to perform Hajj. Hajj can occur in any season, depending on the Islamic (lunar) calendar. It can occur during the summer when the temperature in Makkah can reach 50 °C (Saeed, Schleussner & Almazroui, 2021). This number of pilgrims from different ethnicities, age groups, underlying medical conditions, and other factors, crowding within well-defined geographic boundaries, and moving together can always result in a greater risk of heat-related illnesses (Yezli et al., 2024). Therefore, heatstroke is endemic during Hajj pilgrimage in the desert climate of Saudi Arabia. To mitigate this risk, Saudi authorities have implemented a comprehensive heat-stroke prevention plan for Hajj pilgrims that combines infrastructure, medical preparedness (Al Kassabi & Abed, 2018), and public education (Almuzaini et al., 2022). The Ministry of Hajj and Umrah and Hajj Awareness Centre installed over 400 misting and cooling stations, shaded rest areas, cold-water dispensers, and air-conditioned tents in Mina, Arafat, and around the Grand Mosque, and they applied heat-reflective “white roads” to lower ground temperatures (Aljuhani & Dayem, 2022). The Ministry of Health deployed 34 field units equipped with emergency beds and trained medical teams, launched a Hajj Health Awareness Kit in eight languages, and urged pilgrims to use umbrellas, wear light-colored clothing, drink water every 15–20 min, and avoid direct sun exposure from 10 a.m. to 4 p.m (Al-Khattabi et al., 2024). Timely and effective management is critical for improving the survival rate and heatstroke prognosis (Tobaiqy et al., 2021). The primary intervention is rapid cooling (Comp et al., 2025). The patient should be cooled onsite before transport. The gold standard treatment is immersion in cold water or a similar method until the patient’s core temperature reaches 38.9 °C. Evidence indicates that prompt symptom recognition and implementation of appropriate rapid cooling techniques substantially improve survival outcomes (Chen et al., 2023; Comp et al., 2025).

Health volunteers during the Hajj pilgrimage constitute a critical frontline component in public health strategies aimed at mitigating the incidence of heatstroke among the pilgrim population. Health volunteers must possess the knowledge and skills to act decisively during a life-threatening emergency. Studies suggest that early detection and management of heatstroke reduce the progression of organ damage and reduce mortality (Rublee et al., 2021). There is insufficient research assessing heatstroke recognition and practical intervention knowledge specifically among health volunteers serving during Hajj pilgrimage. Thus, the primary purpose of this study was to assess heatstroke management knowledge among health volunteers during Hajj and identify predictors of their knowledge. We hypothesize that health volunteers with formal heat-illness training, greater years of service during Hajj, and higher educational attainment will demonstrate significantly greater knowledge of heatstroke signs, risk factors, and appropriate first-aid interventions than volunteers without such training or experience.

## Materials & Methods

### Research design

This study employed a descriptive, correlational, quantitative, cross-sectional design using nonprobability-based convenience sampling to assess knowledge of heatstroke among health volunteers in the 2024 Hajj season in the Kingdom of Saudi Arabia.

### Participants/sampling

The targeted population for this study consisted of health volunteers who served and volunteered in Hajj season 2024 from all regions in the Kingdom of Saudi Arabia. The required sample size was calculated *via* published data from the Ministry of Health, which reported a total of 2,838 registered and verified volunteers during Hajj 2024.

The minimum sample size was calculated *via* Cochran’s formula for finite populations (Chaokromthong & Sintao, 2021)

**Figure 1:**
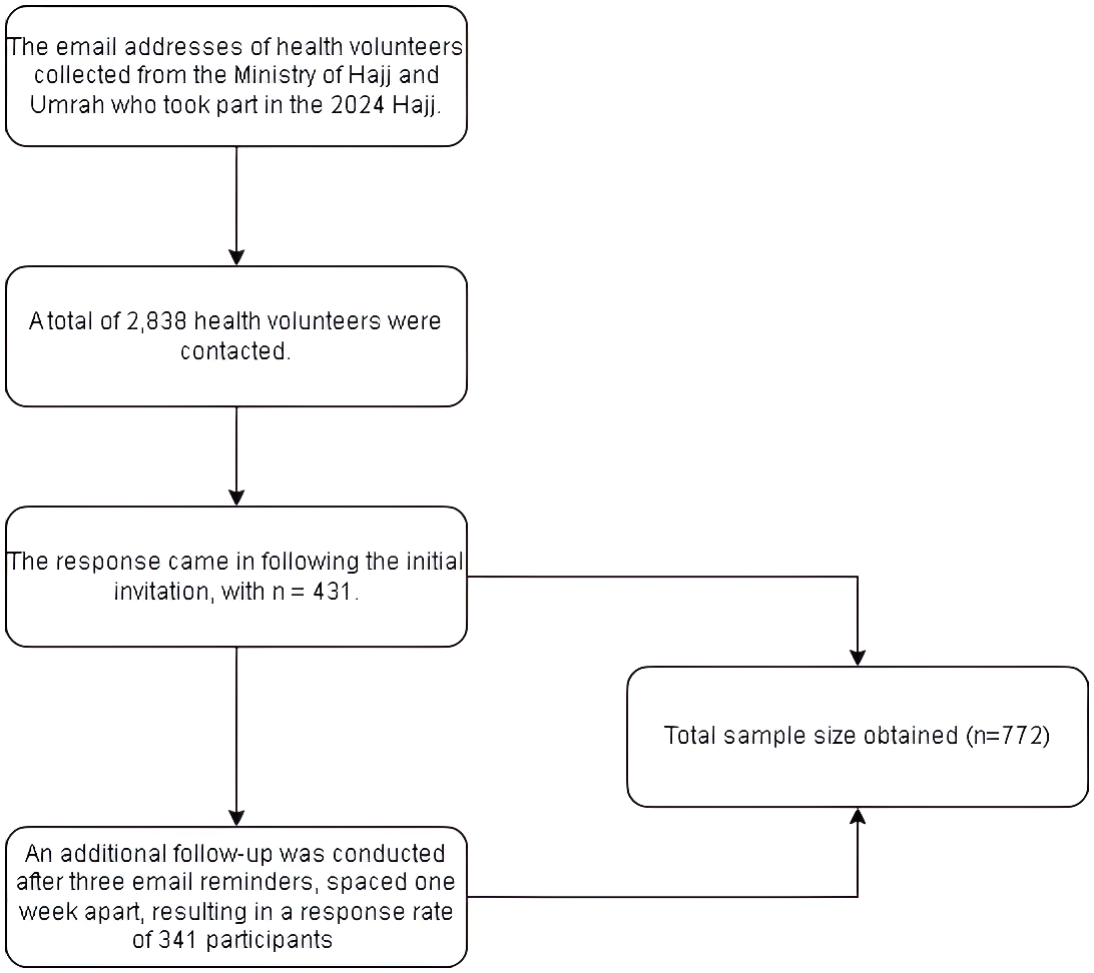
Flow diagram of participant recruitment.

\begin{eqnarray*}n= \frac{Np \left( 1-p \right) }{ \left( N-1 \right) \left( \frac{E}{Z} \right) 2+p(1-P)} \end{eqnarray*}

where *N* is the population size (2,838 registered volunteers), *Z* is the Z score for the 99% confidence level (2.576), *E* is the margin of error (0.05), and p is the estimated proportion (0.5, ensuring maximum variability). This calculation yielded a minimum required sample of 538 participants. To increase statistical power, account for potential nonresponses, and ensure robust subgroup analysis, the study aimed to enroll a larger sample. A total of 2,838 registered health volunteers from the Ministry of Hajj and Umrah were invited to participate. Following the initial invitation, 431 individuals responded (15.2% initial response rate). Subsequent follow-up contact *via* three email reminders spaced one week apart yielded an additional 341 participants, for a total final sample of 772 (27.2% overall response rate) (Fig. 1).

### Data collection

Data collection began after ethical approval was received from the Institutional Review Board at Prince Sultan Military College of Health Sciences. Before data collection, participants were contacted through their registered phone numbers and invited to participate after being informed of the study’s purpose, their voluntary involvement, and their right to withdraw at any time without consequences. The predeveloped 10-minute survey, created *via* Google Forms, was then distributed electronically through social media platforms (WhatsApp group) among the volunteers. Informed consent was obtained online before participation. Data collection took place between June and December 2024.

### Ethical consideration

Ethical approval for this study was obtained from the Institutional Review Board (IRB) of Prince Sultan Military College of Health Sciences (Approval No: IRB-2024-NUR-021). Informed consent was obtained from all participants before their inclusion in the study. The research was conducted in full compliance with relevant ethical guidelines and regulations. Participant anonymity and data confidentiality were strictly maintained throughout the study; no personal identifiers were collected, and all the responses were securely stored and used solely for research purposes.

### Questionnaires development

To assess knowledge of heatstroke among health volunteers during the Hajj season of 2024 in Saudi Arabia, a questionnaire was specifically developed by the research team on the basis of relevant literature, evidence-based references, and official health websites. A key reference used in its development was the validated tool published by Hirschhorn, DadeMatthews & Sefton (2021), which evaluated heatstroke knowledge and management among emergency medical service providers.

The development of the research instrument was a meticulous, multi-phase process designed to ensure comprehensive coverage of the study’s objectives and cross-cultural validity. Initially, a preliminary 30-item questionnaire was drafted, encompassing key domains critical to the research aims: recognition of heatstroke symptoms and pathophysiology, knowledge of evidence-based prehospital management (*e.g.*, cooling techniques), awareness of preventive measures, and familiarity with risk factors. To establish content and face validity, this draft underwent a formal Delphi expert consensus process involving a panel of eight specialists in emergency medicine, sports medicine, public health, and nursing. Over three iterative rounds, panelists rated each item for relevance, clarity, and essentiality on a structured scale. Items failing to achieve a pre-specified consensus level of 80% agreement were systematically refined. All 30-item were retained in knowledge scale. The finalized English version was then translated into Arabic following a standardized protocol of forward- and back-translation to ensure linguistic and conceptual equivalence. Two independent bilingual translators performed the forward translation, the outputs of which were synthesized into a single Arabic version. This version was then back-translated into English by a separate translator blinded to the original instrument. An expert committee, including the principal investigator and a linguist, reviewed all versions to resolve discrepancies, ensuring the final Arabic questionnaire was semantically, idiomatically, and conceptually equivalent to the original, thus making it appropriate for administration to the target population. A subsequent, peer-review conducted during the manuscript publication phase led to the removal of two further items: one (Item 9) which pertained to the use of ointments led to decrease in body temperature, a non-evidence-based practice, the inclusion of which could have artificially inflated knowledge scores through correct rejection for the wrong reason, and another (Item 10) due to substantive redundancy with a core concept already captured in Item 4. Consequently, the ultimate analysis was conducted on a validated 28-item scale. For clarity and consistency in reporting the results, the original numerical labels for the questions were retained in the final figure, resulting in a non-sequential but accurate representation (*e.g.*, Q8 followed by Q11) (Supplementary Data File).

The final questionnaire consisted of two parts:

Part I addressed sociodemographic characteristics (nine items).

Part II assessed heatstroke knowledge through 18 true/false and 10 multiple-choice questions designed to evaluate volunteers’ understanding of and ability to recognize and manage heatstroke.

The questionnaire was prepared in both English and Arabic to accommodate the target population of Arabic-speaking volunteers. For content validation, the initial draft was reviewed by three experts in heatstroke from clinical and academic backgrounds. Their feedback was incorporated, and the questionnaire was revised accordingly. A pilot study was conducted with 32 participants to evaluate the tool’s clarity, structure, and applicability, as well as to estimate completion time. Minor revisions were made on the basis of the pilot findings. The knowledge scores were categorized as satisfactory (≥ 60%, representing ≥17 items correct on the 28-item scale) or unsatisfactory (<60%, representing ≤16 items correct). The cut-off score of 60% to differentiate between ‘Satisfactory’ and ‘Unsatisfactory’ knowledge was established based on the following justifications. Our choice was guided by methodologies used in similar studies conducted in the region. For instance, a study by Almuzaini et al. (2021) assessing heat illness knowledge among healthcare workers during Hajj in Saudi Arabia. They used a 0–10 scale threshold with 5 and below indicating for ‘poor’ knowledge, implying that scores above 50% indicated a baseline of understanding. We adopted a slightly more rigorous threshold of 60% to ensure that the ‘Satisfactory’ category represents a clear and meaningful minimum competency. A score below 60% indicates significant gaps in knowledge that could lead to a failure to recognize critical symptoms like confusion or seizures, or to apply life-saving first aid (*e.g.*, rapid cooling). Therefore, we determined that a score of 60% represents the minimum level of knowledge necessary to perform effectively and safely as a volunteer without posing a risk to pilgrims. Test-retest reliability was assessed using Pearson correlation, which indicated strong relative consistency (*r* = 0.839).

The internal consistency of the 28-item knowledge scale was assessed among 772 participants using Cronbach’s alpha, which indicated a good level of reliability, *α* = .842. The intraclass correlation coefficient (ICC) using a two-way mixed-effects model for consistency further supported the scale’s reliability, with an average measure of ICC = .842, 95% confidence interval (CI) [.826–.858]. These results confirm that the scale possesses acceptable and good internal consistency for use in this study.

### Data analysis

All statistical analyses were conducted using Jeffrey’s Amazing Statistics Program (JASP) (Version 0.18.3; University of Amsterdam, Amsterdam, The Netherlands). The internal consistency of the 28-item knowledge scale was assessed using Cronbach’s alpha, which indicated good reliability. Descriptive statistics, including frequencies and percentages, were computed to summarize participants’ sociodemographic characteristics and responses to individual knowledge items. To examine the bivariate associations between sociodemographic variables and the dichotomized knowledge outcome (satisfactory/unsatisfactory), a series of Pearson’s chi-square tests of independence were performed. The primary inferential analysis was a binary logistic regression, which was executed to identify the independent predictors of satisfactory knowledge. The model included all sociodemographic and experiential variables simultaneously. Model fit was assessed *via* a likelihood ratio test (chi-square), and the model’s explanatory power was evaluated using McFadden, Cox & Snell, and Nagelkerke R^2^ measures. Multicollinearity was assessed using the Variance Inflation Factor (VIF).

## Results

A total of 772 Hajj volunteers participated in this study. The sample was nearly evenly distributed by gender (*n* = 388, 50.3% male; *n* = 384, 49.7% female). The majority of participants were aged 25–29 years (*n* = 328, 42.5%), from the Middle Region (*n* = 427, 55.3%), were university students (*n* = 392, 50.8%), and were employed (*n* = 449, 58.2%). Based on the total knowledge score, participants were categorized into two groups: those with satisfactory knowledge (*n* = 576, 74.6%) and those with unsatisfactory knowledge (*n* = 196, 25.3%). Chi-square and independent samples t-tests revealed significant differences between these two groups across several demographic variables. Participants with satisfactory knowledge were significantly more likely to have a medical educational background (65.8% *vs.* 34.2%, *p* < .001), be employed (62.2% *vs.* 37.8%, *p* < .001), and reside in the Middle Region (*p* < .001). A significant association was also found with age group (p = .001), with a larger proportion of the 25–29 years age group falling into the satisfactory knowledge category compared to the unsatisfactory knowledge group (*n* = 67, 34.2%). No significant difference was observed between knowledge groups based on gender (*p* = .674). A comprehensive summary of all demographic characteristics is presented in Table 1.

**Table 1:** Demographic and background characteristics of Hajj volunteers by knowledge category (*N* = 772).

Characteristic	Total sample (*N* = 772)	Satisfactory knowledge (*n* = 576)	Unsatisfactory knowledge (*n* = 196)	*p*-value
**Age Group, n (%)**				0.001
18 to 24 years	155 (20.1)	98 (17.0)	57 (29.1)	
25–29 years	328 (42.5)	261 (45.3)	67 (34.2)	
30–34 years	192 (24.9)	142 (24.7)	50 (25.5)	
35–39 years	59 (7.6)	45 (7.8)	14 (7.1)	
40–44 years	22 (2.8)	20 (3.5)	2 (1.0)	
45 years and above	16 (2.1)	10 (1.7)	6 (3.1)	
**Gender, n (%)**				0.674
Male	388 (50.3)	292 (50.7)	96 (49.0)	
Female	384 (49.7)	284 (49.3)	100 (51.0)	
**Region, n (%)**				<0.001
Middle Region	427 (55.3)	357 (62.0)	70 (35.7)	
Western Region	135 (17.5)	90 (15.6)	45 (23.0)	
Eastern Region	100 (13.0)	60 (10.4)	40 (20.4)	
Northern Region	72 (9.3)	51 (8.9)	21 (10.7)	
Southwest Region	38 (4.9)	18 (3.1)	20 (10.2)	
**Education Level, n (%)**				<0.001
University student	392 (50.8)	322 (55.9)	70 (35.7)	
Diploma	188 (24.4)	126 (21.9)	62 (31.6)	
Bachelor’s degree	97 (12.6)	72 (12.5)	25 (12.8)	
High school	59 (7.6)	33 (5.7)	26 (13.3)	
Postgraduate education	36 (4.7)	23 (4.0)	13 (6.6)	
**Type of Education, n (%)**				<0.001
Medical	472 (61.1)	379 (65.8)	93 (47.4)	
Non-medical	300 (38.9)	197 (34.2)	103 (52.6)	
**Occupational Status, n (%)**				<0.001
Employed	449 (58.2)	358 (62.2)	91 (46.4)	
Unemployed	323 (41.8)	218 (37.8)	105 (53.6)	
**Number of Previous Volunteering, n (%)**				0.036
Two times	287 (37.2)	231 (40.1)	56 (28.6)	
One time	265 (34.3)	195 (33.9)	70 (35.7)	
Three times or more	220 (28.5)	150 (26.0)	70 (35.7)	
**Previous First Aid Course, n (%)**				0.118
No	508 (65.8)	388 (67.4)	120 (61.2)	
Yes	264 (34.2)	188 (32.6)	76 (38.8)	
**Encountered Heat Stroke, n (%)**				0.03
No	604 (78.2)	461 (80.0)	143 (73.0)	
Yes	168 (21.8)	115 (20.0)	53 (27.0)	
**Total Knowledge Score, Mean (SD)**	20.1 (5.0)	22.4 (3.3)	13.3 (2.5)	<0.001^a^
**Knowledge Percentage, Mean (SD)**	71.7 (17.9)	79.9 (11.6)	47.4 (8.9)	<0.001^a^

**Notes.**

Note SD Standard Deviation

*P*-values are from Chi-Squared tests of independence unless otherwise noted.

a *P*-value from Independent Samples *T*-Test.

**Figure 2:**
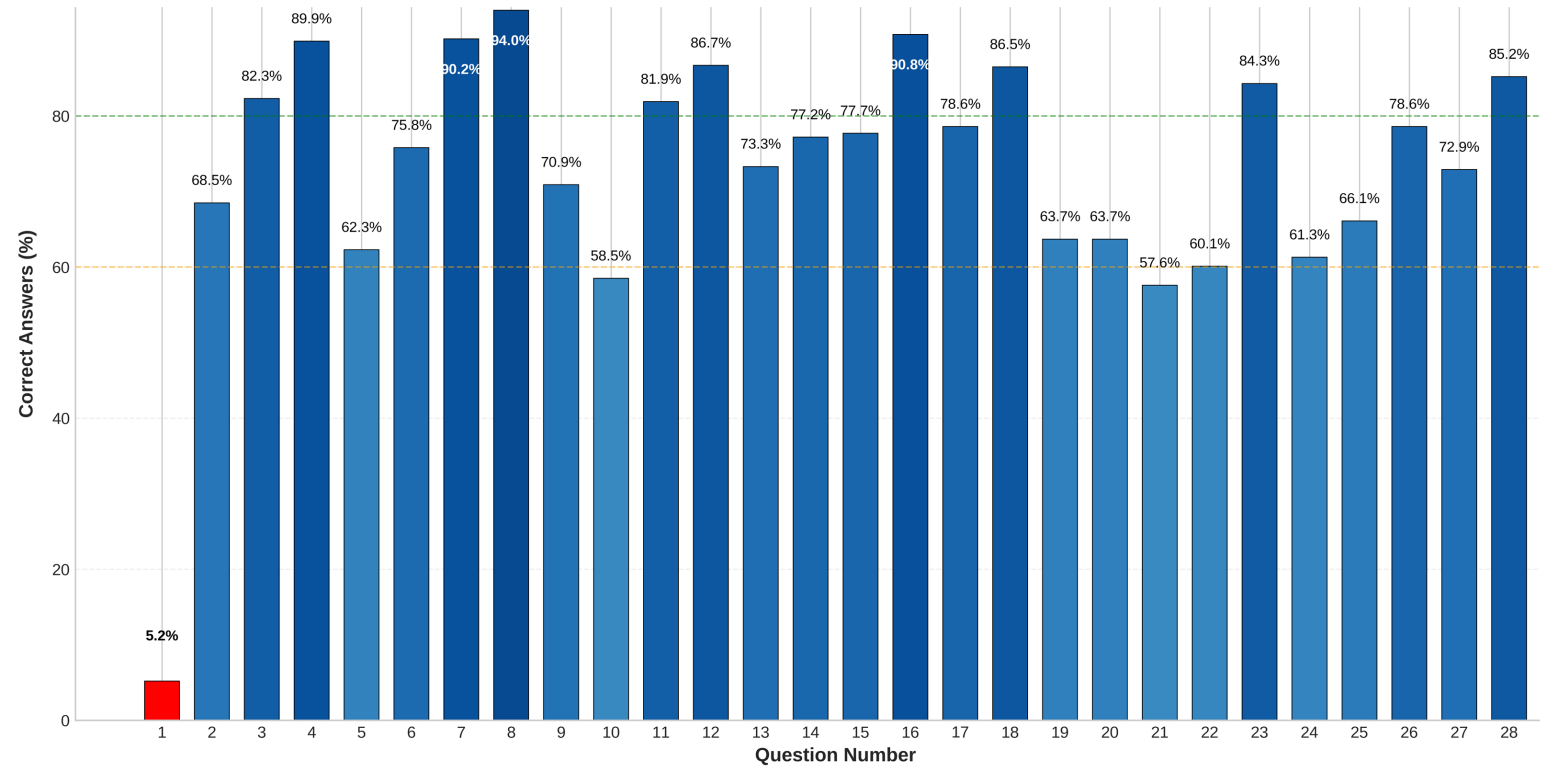
Proportion of correct responses to heatstroke knowledge items among Hajj health volunteers. Question Definitions: Q1: In case of a heat stroke the body temperature will be higher than 38° C; Q2: Shivering reduces body temperature; Q3: Drinking warm/hot fluids is useful to reduce body temperature; Q4: Sweating is instrumental in dissipating excessive heat; Q5: A reduced blood flow to the skin enhances heat dispersal; Q6: An increased blood flow to the skin enhances heat dispersal; Q7: Wearing thinner clothes is useful to maintain low body temperature; Q8: Drinking cold fluids and fresh juice maintains low body temperature; Q9: Heat stroke can be managed using acetaminophen, aspirin and NSAIDs; Q10: In case of high environmental temperatures sweating is always present; Q11: In the elderly sweating may be constitutively impaired; Q12: Walking in hot humid environments may cause severe health complaints; Q13: Heat stroke may follow severe physical activity; Q14: Heat stroke may take place only in warm and humid environments; Q15: Only children and elders are at health risk in case of high temperatures; Q16: Very high body temperatures (*i.e.,* > 39.5 °C) are potentially lethal; Q17: In case of a heat stroke drinking an ‘energy drink’ may be useful; Q18: Exertional heat stroke is considered a life threatening medical emergency; Q19: What is the most accurate method of temperature assessment for the diagnosis; Q20: Rapid cooling of a patient with exertional heat stroke should occur within—minutes from the time of collapse; Q21: Which of the following is the most appropriate cooling method for the management of exertional heat stroke; Q22: Which of the following is correct about the clinical manifestations of exertional heat stroke; Q23: What first aid action should a volunteer take if a heat stroke patient becomes unconscious; Q24: A pilgrim who was in the Arafah complained of a headache and feeling dizzy. The pilgrim’s skin was pale, clammy, and sweaty. This is likely a sign of; Q25: A pilgrim appears to be mentally confused and you are having difficulty understanding what he/she is saying because his/her speech is slurred. The pilgrim’s skin is red and dry, and not sweating. This is likely a sign of; Q26: The basic action to prevent heat stroke for pilgrims is; Q27: What first aid supplies are needed to treat a pilgrim showing signs of heat stroke; Q28: Which of the following foods list that can decrease heat stroke risk.

The analysis of participants’ responses to the knowledge questions about heatstroke management revealed a generally strong understanding, although some misconceptions persisted. A total of 772 participants were assessed, with an overall mean satisfactory knowledge score was 20.1/28 = 71.7% (standard deviation (SD) = 17.9), indicating a solid grasp of the topic (Table 1). The participants demonstrated high accuracy in several areas. Figure 2 shows that (*n* = 726) 94.0% reported that drinking cold fluids and fresh juice helps maintain a low body temperature and (*n* = 726) 90.8% reported that body temperatures exceeding 39.5 °C are potentially lethal. Additionally, (*n* = 694) 89.9% understood the role of sweating in dissipating heat, and (*n* = 692) 90.2% acknowledged that wearing thinner clothes aids in maintaining a low body temperature. Most participants (*n* = 668, 86.5%) also correctly identified exertional heatstroke as a life-threatening emergency, and (*n* = 651) 84.3% knew to check for breathing or heartbeat and begin CPR if necessary for an unconscious heatstroke patient.

However, some misconceptions were evident. Only (*n* = 40) 5.2% correctly reported that a body temperature higher than 38 °C is not always indicative of heatstroke, and (*n* = 243) 31.5% believed that shivering reduces body temperature. While (*n* = 635) 82.3% correctly disagreed that drinking warm or hot fluids is useful for reducing body temperature, (*n* = 137) 17.7% still held this misconception. Furthermore, (*n* = 481) 62.3% answered that reduced blood flow to the skin does not enhance heat dispersal, although (*n* = 585) 75.8% correctly identified increased blood flow as the true mechanism. The participants performed well in identifying clinical scenarios and preventive measures. For example, (*n* = 473) 61.3% correctly recognized pale, clammy, and sweaty skin as signs of heat exhaustion, whereas (*n* = 510) 66.1% identified red, dry skin and mental confusion as indicators of heatstroke. Most participants (*n* = 607) 78.6% emphasized the importance of rest breaks in shaded areas for preventing heatstroke and (*n* = 658) 85.2% correctly identified watermelon, cucumber, and buttermilk as foods that reduce heatstroke risk. In terms of treatment, (*n* = 492) 63.7% of participants accurately identified rectal temperature assessment as the most reliable method for diagnosing exertional heatstroke and (*n* = 445) 57.6% recognized cold-water immersion as the most appropriate cooling method. Additionally, (*n* = 464) 60.1% correctly identified central nervous system (CNS) dysfunction as a key clinical manifestation of exertional heatstroke.

A logistic regression analysis was conducted to model the predictors of satisfactory knowledge levels. The full model, incorporating all predictor variables, demonstrated a statistically significant improvement in fit over the null model, *χ*^2^(20) = 144.27, *p* < .001. The model’s explanatory power, as indicated by several pseudo-R^2^ measures, was modest yet substantive, with McFadden *R*^2^ = 0.165, Cox & Snell *R*^2^ = 0.170, and Nagelkerke *R*^2^ = 0.251. Examination of the coefficients revealed several statistically significant predictors. Specifically, participants aged 40-44 years had odds of satisfactory knowledge 10.057 times greater than the reference age group (Wald *χ*^2^(1) = 7.47, *p* = .009; 95% CI [1.797–56.28]). Educational attainment was a potent predictor; university students (OR = 4.305, Wald *χ*^2^(1) = 19.168, *p* < .001; 95% CI [2.181–8.497]) and those with a bachelor’s degree (OR = 4.510, Wald *χ*^2^(1) = 10.973, *p* = .002; 95% CI [1.778–11.442]) showed significantly elevated odds. Conversely, having a non-medical educational type substantially reduced the odds of satisfactory knowledge (OR = 0.222, Wald *χ*^2^(1) = 21.473, *p* < .001; 95% CI [0.118–0.419]). Employment status (OR = 2.447, Wald *χ*^2^(1) = 12.049, *p* < .001; 95% CI [1.500–3.991]) and previous first aid course exposure (OR = 2.512, Wald *χ*^2^(1) = 8.981, p = .003; 95% CI [1.368–4.614]) were also significant positive predictors. Multicollinearity diagnostics indicated no critical issues, with all VIF values below 3.103 (Table 2).

**Table 2:** Logistic regression analysis predicting odds of satisfactory knowledge.

**Predictor**	**Estimate**	**Robust SE**	**Odds ratio**	**95% CI for OR**	**Wald statistic**	***p* value**
**Intercept**	−0.534	0.528	0.586	[0.208, 1.650]	1.181	0.312
**Gender (Ref: Male)**						
Female	−0.171	0.197	0.843	[0.573, 1.240]	0.703	0.386
**Age Group (Ref: 18–24 years)**						
25–29 years	0.259	0.28	1.296	[0.749, 2.241]	0.864	0.354
30–34 years	−0.022	0.332	0.978	[0.510, 1.877]	0.005	0.948
35–39 years	0.712	0.513	2.038	[0.745, 5.574]	2.35	0.166
40–44 years	2.308	0.879	10.06	[1.797, 56.280]	7.47	**0.009**
45 years and above	0.351	0.761	1.42	[0.320, 6.304]	0.3	0.645
**Region (Ref: Western Region)**						
Middle Region	0.761	0.326	2.141	[1.130, 4.056]	6.211	**0.019**
Eastern Region	−0.499	0.336	0.607	[0.315, 1.173]	2.405	0.137
Northern Region	0.196	0.421	1.216	[0.533, 2.777]	0.235	0.642
Southwest Region	−1.157	0.406	0.314	[0.142, 0.697]	6.956	**0.004**
**Education Level (Ref: High School or less)**						
Diploma	0.423	0.358	1.526	[0.757, 3.075]	1.463	0.237
University student	1.46	0.347	4.305	[2.181, 8.497]	19.168	**<.001**
Bachelor’s degree	1.506	0.475	4.51	[1.778, 11.442]	10.973	**0.002**
Postgraduate education	−0.016	0.611	0.984	[0.297, 3.260]	0.001	0.979
**Type of Education (Ref: Medical)**						
Non-medical	−1.504	0.324	0.222	[0.118, 0.419]	21.473	**<.001**
**Occupational Status (Ref: Not Employed)**						
Employed	0.895	0.25	2.447	[1.500, 3.991]	12.049	**<.001**
**Previous Volunteering (Ref: One time or less)**						
Two times	0.342	0.234	1.408	[0.889, 2.229]	2.029	0.145
Three times or more	−0.14	0.245	0.869	[0.538, 1.404]	0.327	0.566
**Previous First Aid Course (Ref: No)**						
Yes	0.921	0.31	2.512	[1.368, 4.614]	8.981	**0.003**
**Encountered Heatstroke (Ref: No)**						
Yes	0.137	0.262	1.147	[0.686, 1.918]	0.281	0.601

**Notes.**

Note. *N* = 772. The outcome variable is ‘Satisfactory Knowledge’ (coded as 1). Model Fit: *χ*^2^(20) = 144.27, *p* < .001. McFadden’s *R*^2^ = 0.165, Nagelkerke’s *R*^2^ = 0.251. All Variance Inflation Factors (VIF) were below 3.2, indicating no substantial multicollinearity.

SE Standard Error CI Confidence Interval. Statistically significant *p*-values (<.05) are highlighted in bold

## Discussion

This study assessed heatstroke knowledge among health volunteers during the 2024 Hajj pilgrimage in Saudi Arabia, revealing that three-quarters of participants (74.6%, n=576/772) demonstrated satisfactory knowledge (60% and above), with a mean score of 20.1 out of 28. While this finding is encouraging given the critical role these volunteers play in preventing heat-related morbidity and mortality during mass gatherings in extreme environmental conditions, the one-quarter of volunteers (*n* = 196, 25.3%) with unsatisfactory knowledge represents a concerning gap requiring targeted intervention. Multivariate logistic regression identified several independent predictors of knowledge competency, including medical training, educational attainment, geographic region, employment status, and prior first aid training, providing an evidence base for strategic educational initiatives.

In this study health volunteers with medical backgrounds demonstrated substantially higher odds of satisfactory knowledge compared to those without medical training. This was an expected finding as medical curricula adequately address heat-related illness. However, the markedly lower performance among non-medically trained volunteers exposes a critical vulnerability in the volunteer workforce, indicating an urgent need for comprehensive foundational training prior to deployment during Hajj. This knowledge gap can be effectively addressed with recently introduced Integrated Health Volunteering framework, which aims to optimize rapid response systems through enhanced training protocols and artificial intelligence-supported decision-making algorithms (Alharthi & Tounsi, 2025). Despite such innovations, existing evidence suggests substantial deficiencies in pre-deployment preparation. Alrashdi & Al Thobaity (2024) documented that only 44.6% of nurse volunteers received pre-volunteering training for Hajj 2022, highlighting systemic weaknesses in volunteer preparation that must be addressed to ensure effective emergency response capacity. Similar concerns were reported in China were nurses expressed dissatisfaction with their heat illness-related knowledge and skills (Zhao, Lin & Zang, 2021).

Educational level emerged as a powerful predictor of heatstroke knowledge in our study. Health volunteers with university degrees or bachelor’s qualifications exhibited more than four-fold higher odds of satisfactory knowledge compared to those with high school education or less. This association likely reflects critical thinking skills, health literacy, and information exposure associated with tertiary education. Similar to a study conducted by Alsaleh et al. (2025), who identified higher education as an independent predictor of better knowledge about health services among Hajj pilgrims. The consistency across different populations both volunteers and pilgrims underscores the fundamental role of educational attainment in health knowledge acquisition, regardless of specific roles or contexts within the Hajj environment.

Xu et al. (2024) reported similar patterns in China’s general population, where higher education levels correlated with improved knowledge, attitudes, and practices regarding heatstroke. Notably, their study revealed a disconnect between theoretical knowledge and practical application, suggesting that while education enhances understanding, additional interventions may be necessary to translate knowledge into effective field practices. This observation has important implications for volunteer training programs, which should incorporate not only theoretical content but also skill-based and practical learning.

Geographic disparities in knowledge were evident, with volunteers from the Middle Region demonstrating significantly higher mean scores than those from other regions, while volunteers from the Southwest Region scored lowest. These regional differences may reflect variations in training program quality, differential access to health information resources, or baseline differences in public health campaign exposure. The predominance of Middle Region participants in the sample (over half) may also contribute to observed differences, suggesting potential selection effects that warrant consideration.

Age of health volunteers showed a notable though imprecise association, with the 40–44-year age group showing ten-fold increased odds of satisfactory knowledge. While the wide confidence interval 95% CI of OR [1.797–56.280] necessitates cautious interpretation, this finding suggests potential cumulative effects of maturity, experience, and repeated exposure to Hajj-specific health activities. Such age-related knowledge accumulation highlights the value of experienced volunteers in mentoring roles. Similarly, employment status emerged as another significant predictor, with employed volunteers demonstrating more than double the odds of satisfactory knowledge compared to unemployed counterparts.

Prior first aid training showed a strong positive association with heatstroke knowledge, providing empirical evidence that such training effectively builds not only immediate practical skills but also the knowledge for managing medical emergencies. This finding supports the expansion of mandatory first aid certification for all health volunteers regardless of medical background. The importance of structured training and professional development is further underscored by Alshammari, Alyousefi & Almeneessier (2025) systematic review of health volunteerism in Saudi Arabia. They identified several essential interventions such as implementing structured training programs, enhancing institutional support mechanisms, embedding volunteer opportunities within medical curricula, providing financial assistance, establishing comprehensive safety protocols, adopting technology-enabled solutions, aligning with international standards, and ensuring long-term sustainability. These recommendations align with Saudi Vision 2030 objectives and offer a roadmap for strengthening volunteer infrastructure and safe pilgrims journey.

The knowledge levels observed in this study contrast markedly with findings from emergency medical services personnel in other contexts. Hirschhorn, DadeMatthews & Sefton (2021) reported that emergency medical services (EMS) providers scored only 37% correct on exertional heatstroke knowledge assessments, with merely 8.9% having read consensus statements on prehospital exertional heat stroke (EHS) management. Despite over half reporting prior experience treating EHS patients, the majority felt comfortable recognizing and managing these cases, revealing a concerning disconnect between perceived competence and actual knowledge. However, the high knowledge scores observed among Hajj health volunteers in the present study were based on only 28 general questions, which cannot be compared to the comprehensive knowledge assessments that EMS providers undergo. Nevertheless, further enhancing the knowledge and skill level of Hajj health volunteers would represent a valuable addition to future investigations. Equipment accessibility represents another critical dimension of emergency preparedness. Hirschhorn, DadeMatthews & Sefton (2021) documented that only 10% of EMS providers had access to rectal thermometers the gold standard for heatstroke diagnosis with most relying on less effective cooling methods such as chemical cold packs and air conditioning rather than recommended cold-water immersion. While the present study did not assess resource availability, the higher knowledge scores may reflect better equipment access during Hajj, though this hypothesis requires empirical verification. Yezli et al. (2023) systematic review of heatstroke clinical characteristics among Hajj pilgrims documented the severity of this condition, with mean core temperatures of 42.0 °C, widespread early multiorgan injury, and complications including hypotension, acute kidney injury, and coagulopathy. Obesity, diabetes, and cardiovascular disease were prevalent among patients, indicating heightened vulnerability in these populations. Mortality was 5.6%, emphasizing the critical importance of early recognition and aggressive cooling therapy competencies that depend fundamentally on adequate volunteer knowledge.

### Study limitations

Several methodological limitations warrant acknowledgment. The cross-sectional design precludes causal inference and assessment of knowledge trajectories over time. Longitudinal designs would strengthen understanding of how training interventions influence knowledge acquisition and retention. Self-report questionnaire data may have introduced social desirability bias or help-seeking behavior during survey completion, potentially inflating knowledge scores. More rigorous assessment methods, such as scenario-based practical evaluations, would provide more robust competency measures. The sampling strategy represents the most significant limitation. Convenience sampling of more than half the volunteers, combined with over-representation of Middle Region participants, limits generalizability across Saudi Arabia. Future investigations should employ stratified random sampling with regional quotas to ensure balanced geographic representation and enhance external validity. Additionally, the wide confidence interval for the 40–44-year age group association suggests limited precision in age-related estimates, necessitating larger sample sizes in specific age strata.

### Conclusions

Approximately three-quarters of Hajj health volunteers demonstrated satisfactory heatstroke knowledge (mean = 71.7%, SD = 17.9%), though the one-quarter with unsatisfactory knowledge represents a significant gap. Medical training, educational attainment, geographic region, and employment status were independent predictors of knowledge competency. These findings demonstrate that knowledge is not uniformly distributed. Targeted interventions should prioritize non-medical volunteers and underperforming regions to ensure consistent preparedness across the volunteer workforce.

### Recommendations

First, develop evidence-based training modules addressing physiological concepts and practical cooling techniques, with regional workshops tailored to underperforming areas. Second, prioritize non-medical volunteers through targeted foundational training. Third, integrate simulation-based learning and practical drills to enhance hands-on competency. Finally, ensure equitable resource distribution and conduct regular knowledge assessments to measure training effectiveness.

##  Supplemental Information

10.7717/peerj.20816/supp-1 Supplemental Information 1 Questionnaire used in the research

10.7717/peerj.20816/supp-2 Supplemental Information 2 Raw dataset

## Additional Information and Declarations

### Data Availability

The following information was supplied regarding data availability: The raw data are available in the Supplemental File.
