# Supplementary material for: Heatstroke knowledge and predictors among Hajj health volunteers in Saudi Arabia: a cross-sectional study
Source: PeerJ. 2026 Feb 19;14:e20816. doi: 10.7717/peerj.20816 (PMC12925418; doi:10.7717/peerj.20816)
Supplement: Supplemental Information 1 [file peerj-14-20816-s001.pdf]

	**Prince Sultan Military College for Health Science**<br>**Scientific Research**<br><br>**Assessing Heat Stroke Management Knowledge Among Health Volunteers in Hajj in KSA and Identifying Predictors**	**كلية المير سلطان العسكرية للعلوم الصحية بحث علمي بعنوان**<br><br>**تقييم المعرفة المتعلقة بإدارة ضربات الشمس بين المتطوعين الصحيين في الحج بالمملكة العربية السعودية وتحديد عوامل التنبؤ بها**
	**Section one (1): Socio-demographic characteristics:**	**القسم الول (1): الخصائص الجتماعية والديموغرافية:**
1	**Gender:**<br>A. Male<br>B. Female	**الجنس:**<br>أ.ذكر<br>ب.أنثى
2	**Age:**<br>A. 18-24 years<br>B. 25-29 years<br>C. 30-34 years<br>D. 35-39 years<br>E. 40-44 years<br>F. 45-49 years<br>G. 50 years or more	**العمر:**<br>أ. 18-24 سنة<br>ب.25-29 سنة<br>ت.30-34 سنة<br>ث. 35-39 سنة<br>ج.40-44 سنة د.<br>45-49 سنة<br>خ. 50 سنة أو أكثر
3	**Region:**<br>A. East<br>B. West<br>C. North<br>D. South<br>E. Middle	**المنطقة:**<br>أ. لشرقية ب.<br>الغربية ت.<br>الشمال ث.<br>الجنوب ج.<br>الوسط
4	**Education level:**<br>A. High school<br>B. Diploma<br>C. University student<br>D. Bachelor’s degree<br>E. Postgraduate education	**مستوى التعليم:**<br>أ. المدرسة الثانوية ب.<br>شهادة دبلوم ت.<br>طالب جامعي<br>ث. درجة البكالوريوس ج.<br>دراسات عليا
5	**Type of education:**<br>A. Medical<br>B. Non-medical	**نوع التعليم:**<br>A. طبي<br>B. غير طبي
6	**Occupational status:**<br>A. Employed<br>B. Unemployed	**الوضع المهني:**<br>أ.موظف ب.غير<br>موظف
7	**Number of previous volunteering:**<br>A. One	**عدد العمال التطوعية السابقة:**<br>أ. واحد

	B. Two<br>C. Three or more	ب. اثنين<br>ت. ثلثة أو أكثر
8	**Previous exposure to first aid course:**<br>A. Yes<br>B. No	**التعرض السابق لدورة السعافات الولية:**<br>أ. نعم<br>ب. ل
9	**Have you encountered a heat stroke situation?**<br>A. Yes<br>B. No	**هل تعاملت سابقا مع حالة ضربة الشمس؟**<br>أ. نعم<br>ب. ل
	**Section two (2): General knowledge test**<br>**I. Please select "T" if the statement is true and "F" if it is false.**	**القسم الثاني (2): اختبار المعرفة العامة**<br>**I.يرجى اختيار "T" إذا كانت العبارة صحيحة و "F"إذا كانت خاطئة.**
1	In case of a heat stroke the body's temperature will be higher than 38 C. **False**	في حالة الصابة بضربة الشمس، ستكون درجة حرارة الجسم أعلى من 38 درجة مئوية.
2	Shivering reduces body temperature. **True**	الرتعاش يخفض درجة حرارة الجسم.
3	Drinking warm/hot fluids is useful to reduce body temperature. **False**	شرب السوائل الدافئة/الساخنة مفيد لخفض درجة حرارة الجسم.
4	Sweating is instrumental in dissipating excessive heat. **True**	التعرق له دور فعال في تبديد الحرارة الزائدة.
5	A reduced blood flow to the skin enhances heat dispersal. **False**	انخفاض تدفق الدم إلى الجلد يعزز تشتت وتخفيض الحرارة.
6	An increased blood flow to the skin enhances heat dispersal. **True**	زيادة تدفق الدم إلى الجلد يعزز تشتت وتخفيض الحرارة.
7	Wearing thinner clothes is useful to maintain low body temperature. **True**	ارتداء الملبس الرقيقة مفيد للحفاظ على درجة حرارة الجسم منخفضة.
8	Drinking cold fluids and fresh juice maintains low body temperature. **True**	شرب السوائل يحافظ على انخفاض درجة حرارة الجسم.
9	Heat stroke can be managed using acetaminophen, aspirin, and NSAIDs. **False**	يمكن إدارة ضربة الشمس باستخدام السيتامينوفين والسبرين ومضادات اللتهاب غير الستيروئيدية.

10	In case of high environmental temperatures, sweating is always present. **False**	في حالة ارتفاع درجات الحرارة في الجو، يكون التعرق موجودا دائما.
11	In the elderly, sweating may be constitutively impaired. **True**	عند كبار السن، قد يكون التعرق ضعيفا بشكل واضح.
12	Walking in hot, humid environments may cause severe health complaints. **True**	المشي في الجو الحار والرطب قد يسبب مشاكل صحية خطيرة.
13	Heat stroke may follow severe physical activity. **True**	قد تأتي ضربة الشمس بعد نشاط بدني شديد.
14	Heat stroke may take place only in warm and humid environments. **False**	قد تحدث ضربة الشمس فقط في الجواء الدافئة والرطبة.
15	Only children and elders are at health risk in case of high temperatures. **False**	الطفال وكبار السن فقط هم المعرضون للخطر الصحي وضربات الشمس في حالة ارتفاع درجات الحرارة.
16	Very high body temperatures (i.e. > 39.5°C) are potentially lethal. **True**	من المحتمل أن تكون درجات حرارة الجسم المرتفعة جدا مميتة (أي< 39.5 درجة مئوية).
17	In case of a heat stroke, drinking an "energy drink" may be useful. **False**.	في حالة الصابة بضربة الشمس، قد يكون شرب "مشروب الطاقة" مفيدا.
18	Exertional heat stroke is considered a life-threatening medical emergency. **True**	تعتبر ضربة الشمس الناتجة عن الجهاد حالة طبية طارئة تهدد الحياة.
	**II. Please select the circle around the correct answer only.**	**ثانيا. الرجاء تحديد الدائرة حول الجابة الصحيحة فقط.**
19	What is the most accurate method of temperature assessment for the diagnosis of exertional heat stroke in the prehospital setting?<br>a) Oral.<br>b) Tympanic.<br>c) Axillary.	ما هي الطريقة الكثر دقة لقياس درجة الحرارة لتشخيص ضربة الشمس الجهدية في بيئة ما قبل المستشفى؟<br>أ. الفم.<br>ب. الذن.<br>ت. البط.<br>ث. الشرج.
	**d) Rectal.**	

20	Rapid cooling of a patient with exertional heat stroke should occur within ____minutes from the time of collapse.<br>a) 15<br>**b) 30**<br>c) 45<br>d) 60	يجب أن يحدث التبريد السريع للمريض المصاب بضربة الشمس خلل____دقيقة من وقت النهيار.<br>أ. 15<br>ب.30<br>ت.45<br>ث.60
21	Which of the following is the most appropriate cooling method for the management of exertional heat stroke?<br>**a) Cold-water immersion from the neck down.**<br>b) Ice packs on the whole body.<br>c) Fanning the patient.<br>d) Providing the patient with oral fluids for rehydration.	أي مما يلي هو أسلوب التبريد الكثر ملءمة لدارة ضربة الشمس الناتجة عن الجهد؟<br>أ.التغطيس في الماء البارد من الرقبة إلى السفل. ب. وضع كمادات من الثلج على كامل الجسم. ت. تهوية المريض.<br>ث. تزويد المريض بالسوائل عن طريق الفم لمعالجة الجفاف.
22	Which of the following is correct about the clinical manifestations of exertional heat stroke?<br>a) Hot, dry skin<br>b) Cool, clammy skin<br>**c) CNS dysfunction (e.g., confusion, altered mental status)**<br>d) Vomiting, profuse sweating, dehydration	أي مما يلي صحيح فيما يتعلق بالعلمات السريرية لضربة الشمس الناتجة عن الجهد؟<br>أ. الجلد الساخن والجاف ب.<br>بشرة باردة ورطبة<br>ت. خلل في الجهاز العصبي المركزي (مثل الرتباك وتغير الحالة العقلية)<br>ث. القيء، والتعرق الغزير، والجفاف
23	What first aid action should a volunteer take if a heat stroke patient becomes unconscious?<br>**a) Check for signs of breathing or heartbeat and begin CPR if there are none.**<br>b) Immediately begin chest compressions at a rate of 120 compressions per minute.<br>c) Attempt to give the patient small sips of water.<br>d) Wipe down the skin with alcohol wipes if available.	ما هي إجراءات السعافات الولية التي يجب على المتطوع القيام بها إذا أصبح مريض ضربة الشمس فاقدا للوعي؟<br>أ. تحقق من وجود علمات التنفس أو ضربات القلب وابدأ في النعاش القلبي الرئوي إذا لم تكن هناك علمات.<br>ب. ابدأ على الفور بالضغط على الصدر بمعدل 120 ضغطة في الدقيقة.<br>ت. حاول إعطاء المريض رشفات صغيرة من الماء. ث. امسح الجلد بمناديل الكحول إن وجدت.

24	A pilgrim, who was in the Arafah, complained of a headache and feeling dizzy. The pilgrim's skin was pale, clammy, and sweaty. This is likely a sign of:<br><br>a) Heat stroke.<br>b) Heat cramps.<br>**c) Heat exhaustion.**<br>**d)** Heat syncope.	شكا أحد الحجاج وهو في عرفة من صداع ودوار. كان جلد الحاج شاحبا ورطبا ومتعرقا. من المحتمل أن تكون هذه علمة على:<br>أ.ضربة شمس.<br>ب. تشنجات حرارية. ت. النهاك<br>الحراري. ث. إغماء الحرارة.
25	A pilgrim appears to be mentally confused, and you are having difficulty understanding what he/she is saying because his/her speech is slurred. The pilgrim's skin is red and dry, and not sweating. This is likely a sign of:<br>**a) Heat stroke.**<br>b) Heat cramps.<br>c) Heat exhaustion.<br>d) Heat syncope.	وجدت ان الحاج كان مشوشا ذهنيا، وتواجه صعوبة في فهم ما يقوله بسبب تشويش كلمه. وكان جلد الحاج أحمر جافا ل يتعرق. من المحتمل أن تكون هذه علمة على:<br>أ.ضربة شمس.<br>ب. تشنجات حرارية. ت.<br>النهاك الحراري. ث.<br>إغماء الحرارة.
26	The basic action to prevent heat stroke for pilgrims is:<br>a) Drinking a cup of cool water every three hours to stay hydrated.<br>**b) Taking rest breaks in a shaded area or under an umbrella.**<br>c) Drinking tea and caffeine during the day.<br>d) Eating foods that are high in carbohydrates and sugar.	الجراء الساسي للوقاية من ضربة الشمس للحجاج هو:<br>أ. شرب كوب من الماء البارد كل ثلث ساعات للبقاء رطبا. ب. أخذ فترات راحة في منطقة مظللة أو تحت المظلة.<br>ت. شرب الشاي والكافيين خلل النهار.<br>ث. تناول الطعمة التي تحتوي على نسبة عالية من الكربوهيدرات والسكر.
27	What first aid supplies are needed to treat a pilgrim showing signs of heat stroke?<br>a) A block or stand for putting up the pilgrim's feet above heart level.<br>b) Ice packs or cold packs for placement in the arm pits and groin areas.<br>c) A spray bottle with water and fans.<br>d) **All of the above.**	ما هي لوازم السعافات الولية المطلوبة لعلج الحاج الذي تظهر عليه علمات ضربة الشمس؟<br>أ. كتلة أو كرسي لرفع قدم الحاج فوق مستوى القلب.<br>ب. كمادات الثلج أو الكمادات الباردة لوضعها في منطقة الذراعين والفخذ.<br>ت. زجاجة رذاذ بها ماء ومراوح.<br>ث. كل ما ورد اعله.
28	Which of the following foods list that can decrease heatstroke risk?<br>a) Watermelon, Cucumber, Caffeine.<br>b) Buttermilk, Salty foods, Sugary foods.<br>c) Caffeine, Salty foods, Sugary foods.<br>d) **Watermelon, Cucumber, Buttermilk.**	أي من الطعمة التالية يمكن أن تقلل من خطر الصابة بضربة الشمس؟<br>أ. البطيخ، الخيار، الكافيين.<br>ب. اللبن، الطعمة المالحة، الطعمة السكرية. ت. الكافيين،<br>الطعمة المالحة، الطعمة السكرية. ث. البطيخ، الخيار، اللبن.
